# Perioperative Risk Prediction in Head and Neck Free Flap Reconstructive Surgery

**DOI:** 10.1111/aas.70115

**Published:** 2025-08-31

**Authors:** Karolina Persson, Madeleine Torén, Louise Walther Sturesson, Thomas Kander, Caroline Nilsson, Johanna Sjövall

**Affiliations:** ^1^ Department of Intensive and Perioperative Care Skåne University Hospital Lund Sweden; ^2^ Department of Clinical Sciences Lund Lund University Lund Sweden; ^3^ Department of Surgery Helsingborg Hospital Helsingborg Sweden; ^4^ Department of Otorhinolaryngology, Head and Neck Surgery Skåne University Hospital Lund Sweden

## Abstract

**Background:**

Extensive head and neck reconstructive surgery is a complex procedure often performed in patients with multiple comorbidities, and the risk of complications is high. Evidence‐based preoperative assessment and reliable risk prediction are therefore essential, and the anesthesiologist plays an important role in this process. The aim of this study was to evaluate the predictive properties of readily available clinical parameters for postoperative complications.

**Methods:**

We performed a retrospective registry study including 388 patients undergoing head and neck free flap surgery between 2009 and 2022. Logistic regression analyses were used to establish associations between perioperative variables and postoperative flap compromise and systemic complications during primary in‐hospital stay. Perioperative variables included risk prediction instrument scores, biochemical laboratory values, type of flap, surgery time, and fluids and drugs administered.

**Results:**

Factors associated with flap compromise in multivariable analysis were surgery time (*p* = 0.005) and perioperative red blood cell transfusion (*p* = 0.001). American Society of Anesthesiologists Physical Status (ASA‐PS) (*p* = 0.012), Charlson Comorbidity Index (CCI) (*p* = 0.021) and Head Neck Charlson Comorbidity Index (HN‐CCI) (*p* = 0.024) were factors most significantly associated with systemic complications.

**Discussion:**

Strong association was seen between surgery time and perioperative red blood cell transfusion and flap compromise. ASA‐PS, CCI, and its simplified version HN‐CCI were shown to be independently associated with systemic complications. To the best of our knowledge, this is the first study demonstrating the value of Head Neck Charlson Comorbidity Index in this setting.

**Editorial Comment:**

This single center cohort analysis describes factors associated with head and neck free flap surgery postoperative flap compromise and other major complications. Established comorbidity indices were included in the analysis.

## 1 Introduction

Extensive head and neck reconstructive surgery using free flaps is a highly complex surgical procedure with a vast range of potential difficulties. The frequency of complications is high, leading to immense effects on patients, families, and society [1, 2, 3]. In addition, many patients have significant comorbidities and have undergone previous oncologic treatment, further increasing complication risks [4, 5, 6]. Nevertheless, extensive surgery is often the only chance of cure [7]. Prediction of complications—and potentially intervention—prior to the choice of treatment is therefore desirable, but, for lack of reliable predictors, the decisions of perioperative management often rely on expert opinion [8]. While decisions about treatment plans often are made by surgeons and/or oncologists, early involvement of anesthesiologic competence is key for an optimal perioperative process. To facilitate treatment decisions, risk prediction instruments including risk assessment calculators and comorbidity indices, with higher scores indicating multiple or more severe comorbidities, have raised increasing interest. However, the models vary widely in design, patient populations, independent variables, and outcomes, making comparison and application challenging [9].

Developed in the early 1940s, the American Society of Anesthesiologists Physical Status Classification, ASA‐PS, is one of the most used preoperative risk prediction tools worldwide. Although originally not designed for this purpose [10] it has been tested in countless perioperative settings, and the support for its use in conjunction with additional data, i.e., biochemical parameters, seems strong [11]. For the head and neck free flap patient population, several studies have shown high predictive properties for medical and surgical postoperative morbidity as well as mortality [12, 13]. Despite a substantial interrater variability [14] its simplicity has rendered it continuously popular.

Many other risk prediction instruments have followed in the wake of ASA‐PS, developed by different specialties and with focus on more or less specific patient groups and outcomes [15]. For head and neck cancer (HNC) patients, several instruments have been evaluated [16], and attempts have also been made at tailored models for patients requiring free flap reconstruction, although none have gained predominant impact [17, 18, 19].

One widely used model is the Charlson Comorbidity Index (CCI). Originally introduced in the 1980s as a prediction instrument of mortality in hospitalized patients [20], it has also been used in the perioperative setting. For patients undergoing head and neck free flap surgery, its applicability to predict postoperative complications has been shown in several studies [21, 22].

Several modified CCI models also exist, among these the Head Neck Charlson Comorbidity Index (HN‐CCI). It was developed by Bøje et al. [23] to predict survival in patients with head and neck squamous cell carcinoma treated with radiotherapy, and has since its introduction in 2013 shown promising predictive properties for preoperative risk prediction [24]. However, its use for the specific free flap surgery population has, to our knowledge, not been established.

Furthermore, the American College of Surgeons National Surgical Quality Improvement Project (ACS‐NSQIP) risk calculator was presented in the early 21st century as providing individual risks for several postoperative complications after specific surgical procedures, but studies on head and neck free flap patients have shown mixed results [25, 26].

In addition to the different types of risk prediction instruments a variety of single and combined clinical parameters, i.e., biochemical tests, lifestyle factors and intraoperative aspects, have been shown to predict postoperative morbidity and mortality, but no single one has been found superior for all purposes [27, 28, 29]. A “theory of everything”‐risk prediction instrument is likely very difficult to establish due to the aforementioned wide differences in model construction, included variables and outcomes. However, despite its weaknesses, risk prediction is a well‐recognized part of the preoperative patient evaluation and should, with early and close involvement of anesthesiologists, be included in any modern multidisciplinary treatment plan.

This study aimed to identify factors associated with postoperative complications potentially accessible to pre‐ and perioperative interventions by evaluating well‐known preoperative risk prediction instruments combined with basic and readily available clinical parameters in patients undergoing major head and neck surgery, including microvascular reconstruction.

## 2 Patients and Methods

This registry‐based observational study was approved by the Swedish Ethical Review Authority, dnr 2019‐02011, June 12th 2019. The need for written consent was waived by the authority and replaced by an opt‐out possibility. The study is reported in accordance with the STROBE guidelines.

Skåne University hospital is a tertiary referral hospital in the Southern Health Care Region in Sweden, with a catchment area of approximately 1.9 million inhabitants. All consecutive patients subjected to microvascular free flap surgery in the head and neck area at Skåne University Hospital, Sweden, from 2009 to 2022 were included. Patients who used the opt‐out possibility were excluded, as well as patients where access to medical records was blocked for privacy reasons.

### 2.1 Data Extraction

The database was created by manual merging of information from several electronic systems, including electronic medical charts (Melior, Cerner, Munich, Germany), the surgical planning system (Orbit, Tietoevry Care, Helsinki, Finland), the patient data management system at the intensive care unit, ICU, (IntelIispace Critical Care and Anesthesia (ICCA), Phillips, Amsterdam, Netherlands), the ICU quality registry (PasIVA, Otimo Data AB, Kalmar, Sweden) and from scanned paper‐based anesthesiology records.

Variables extracted from electronic systems included baseline patient characteristics, tumor classification, any previous HNC and treatment thereof, and surgical indication, as well as preoperative biochemical laboratory values.

ASA‐PS class was noted, and scores for the comorbidity indices CCI and HN‐CCI were calculated for each patient (for CCI the two points for tumor were deducted). An ACS NSQIP Risk Calculator score was calculated for all patients using the online NSQIP Surgical Risk Calculator with the CPT code: 42894 (resection of pharyngeal wall requiring closure with myocutaneous or fasciocutaneous flap or free muscle, skin, or fascial flap with microvascular anastomosis). All scoring of risk prediction instruments was made by the same researcher (MT), with spot‐checks by other team members.

Furthermore, intra‐ and postoperative variables, including the type of free flap used, duration of surgery, and postoperative biochemical laboratory values, were extracted.

### 2.2 Outcomes

The outcomes were divided into flap compromise and systemic complications during the primary in‐hospital stay. Flap compromise included flap necrosis (total or partial), return to the operating room due to flap insufficiency, and deep surgical site infection, all with a clinical significance corresponding to Clavien‐Dindo grade 2–4b complications. Systemic complication was defined as sepsis, pulmonary embolism, deep vein thrombosis, arrhythmia (atrial fibrillation, atrioventricular blockage or need for rhythm conversion), myocardial infarction, pneumonia, heart failure, return to the ICU, and transfer to internal medicine high dependency unit or cardiac intensive care unit.

### 2.3 Statistics

All continuous variables are presented with median (interquartile range) and all numbers are presented with percent (%). A *p*‐value < 0.05 is considered significant. Ninety‐five percent confidence intervals are given for odds ratios.

Univariate logistic regression analyses were performed for all pre‐, peri‐, and postoperative variables for the two primary outcomes, flap compromise and systemic complications. Multivariable logistic regression analyses were conducted using the Backward Wald method, with only the results from the final step presented. Independent variables were chosen based on the results from the univariate analyses and results from previous studies, with a focus on parameters potentially amenable to future pre‐ and perioperative interventions. Given that the number of each outcome was limited, the number of independent variables in each multivariable logistic regression analysis was limited to a maximum of one per 10 outcomes. Spearman's correlation test was conducted for all significant univariate variables to ensure that collinearity was not substantial, and variables with an *r*‐value of > 0.8 were not included in the same model.

All statistical analyses were performed using SPSS v. 27 (IBM., Chicago, IL, USA).

## 3 Results

A total of 389 patients were eligible for inclusion. One patient had access to medical records blocked for privacy reasons and was therefore excluded, leaving 388 procedures for analysis. In 351 cases (95%) the indication for surgery was tumor (malignant or benign). Other reasons for surgery were trauma or osteoradionecrosis. Flap compromise occurred in 53 cases (14%), and complete flap failure in 25 cases (6%). Forty‐eight patients (12%) suffered from at least one systemic complication during primary hospitalization.

### 3.1 Univariate Analyses

Results of univariate regression analyses of pre‐, peri‐, and postoperative parameters versus both flap and systemic complications are presented in Tables 1 and 2. A summary of the results is presented below.

**TABLE 1:** Univariate analyses of preoperative baseline data, risk prediction instrument scores and biochemical laboratory values.

Variable	All *n* = 388 (100%)	No flap compromise *n* = 335 (86%)	Flap compromise *n* = 53 (14%)	*p* (95% CI of OR)	No systemic complication *n* = 340 (88%)	Systemic complication *n* = 48 (12%)	*p* (95% CI of OR)
Gender							
Male	236 (61%)	208 (62%)	28 (53%)	Reference	208 (61%)	28 (58%)	Reference
Female	152 (39%)	127 (38%)	25 (47%)	0.201 (0.817–2.619)	132 (39)	20 (42%)	0.706 (0.609–2.080)
Missing	0 (0%)						
Age (years)	66 (56–71)	65 (56–71)	68 (56–72)	0.282 (0.990–1.037)	65 (55–71)	68 (60–73)	**0.021 (1.005–1.062)**
Missing	0 (0%)						
Employment status							
Worker/student/retiree	165 (43%)	309 (92%)	48 (91%)	Reference	312 (92%)	45 (94%)	Reference
Unemployed/early retiree	217 (56%)	20 (6%)	5 (9%)	0.363 (0.577–4.490)	22 (7%)	3 (6%)	0.930 (0.272–3.287)
Missing	6 (2%)	6 (2%)	0 (0%)		6 (2%)	0 (0%)	
Civil status							
Single	132 (34%)	118 (35%)	14 (26%)	Reference	114 (34%)	18 (38%)	Reference
Couple	252 (65%)	213 (64%)	39 (73%)	0.191 (0.805–2.958)	222 (65%)	30 (63%)	0.626 (0.457–1.601)
Missing	4 (1%)	4 (1%)	0 (0%)		4 (1%)	0 (0%)	
Children							
Has children	293 (75%)	251 (75%)	42 (80%)	Reference	255 (75%)	38 (80%)	Reference
Has no children	55 (14%)	52 (16%)	3 (6%)	0.084 (0.866–9.714)	47 (14%)	8 (17%)	
Missing	40 (10%)	32 (10%)	8 (15)		38 (11%)	2 (4%)	0.752 (0.384–1.995)
Smoking							
Current smoker	113 (29%)	99 (30%)	14 (26%)	Reference	123 (36%)	9 (19%)	Reference
Non/previous smoker	252 (65%)	216 (65%)	36 (68%)	0.626 (0.438–1.644)	200 (59%)	33 (69%)	0.160 (0.265–1.244)
Missing	23 (6%)	20 (6%)	3 (6%)		17 (5%)	6 (13%)	
Alcohol consumption							
None or moderate	241 (62%)	202 (60%)	39 (74%)	Reference	215 (63%)	26 (54%)	Reference
Overconsumption	53 (14%)	49 (15%)	4 (8%)	0.117 (0.144–1.239)	48 (14%)	5 (10%)	0.771 (0.315–2.358)
Missing	94 (24%)	84 (25%)	10 (19%)		77 (23%)	17 (35%)	
BMI (kg/m^2^)	24 (22–28)	25 (22–28)	23 (21–27)	0.186 (0.892–1.022)	24 (22–28)	24 (22–28)	0.825 (0.943–1.076)
Missing	0 (0%)						
Hypertension							
Yes	146 (38%)	130 (39%)	16 (31%)	0.231 (0.365–1.276)	125 (37%)	21 (44%)	0.351 (0.726–2.466)
Missing	0 (0%)						
Systolic blood pressure							
	141 (130–160)	140 (130–160)	145 (130–160)	0.911 (0.988–1.014)	140 (130–160)	148 (126–161)	1.0 (0.987–1.013)
Missing	0 (0%)						
Diabetes							
Yes	41 (11%)	39 (12%)	2 (4%)	0.102 (0.70–1.271)	33 (10%)	8 (17%)	0.147 (0.803–4.309)
Missing	0 (0%)						
Cardiovascular disease							
Yes	50 (13%)	47 (14%)	3 (6%)	0.104 (0.110–1.227)	43 (13%)	7 (15%)	0.708 (0.498–2.795)
Missing	0 (0%)						
Liver disease							
Yes	1 (0.3%)	1 (0.3%)	0 (0%)	1.0 (—)	1 (0.3%)	0 (0%)	1.0 (—)
Missing	0 (0%)						
COPD							
Yes	18 (5%)	16 (5%)	2 (4%)	0.748 (0.175–3.502)	13 (4%)	5 (10%)	0.051 (0.994–8.607)
Missing	0 (0%)						
Immunosuppression							
Yes	56 (14%)	6 (2%)	2 (4%)	0.356 (0.422–10.945)	8 (2%)	0 (0%)	0.999 (—)
Missing	0 (0%)						
Anticoagulants							
Yes	70 (18%)	62 (19%)	8 (15%)	0.783 (0.351–1.744)	58 (17%)	12 (25%)	0.184 (0.796–3.302)
Missing	0 (0%)						
ASA class							
	2 (2–3)	2 (2–3)	2 (2–3)	0.419 (0.753–1.980)	2 (2–3)	3 (2–3)	**0.002 (1.382–4.032)**
Missing	4 (1%)	4 (1%)	0 (0%)		4 (1%)	0 (0%)	
CCI score							
	2 (1–4)	2 (1–4)	3 (1–4)	0.578 (0.877–1.266)	2 (1–3)	3 (2–4)	**0.005 (1.085–1.588)**
Missing	0 (0%)						
HN‐CCI score							
≥ 1	85 (22%)	74 (22%)	11 (21%)	0.827 (0.456–1.883)	67 (20%)	18 (38%)	**0.006 (1.286–4.648)**
Missing	0 (0%)						
NSQIP (any compl)							
	18% (16%–22%)	18% (16%–22%)	18% (16%–21%)	0.867 (0.930–1.063)	18% (16%–22%)	20% (17%–23%)	0.054 (0.999–1.144)
Missing	0 (0%)						
NSQIP (serious compl)							
	15% (13%–20%)	15% (13%–20%)	16% (14%–19%)	0.717 (0.923–1.056)	15% (13%–20%)	18% (15%–22%)	**0.006 (1.027–1.176)**
Missing	0 (0%)						
Hemoglobin (g/L)							
	137 (126–148)	138 (126–148)	136 (126–142)	0.089 (0.967–1.002)	137 (126–148)	138 (126–146)	0.599 (0.976–1.014)
Missing	0 (0%)						
Albumin (g/L)							
	40 (38–43)	40 (38–43)	42 (38–43)	0.663 (0.947–1.089)	40 (38–44)	39 (37–41)	**0.026 (0.865–0.991)**
Missing	24 (6%)	18 (5%)	6 (11%)		23 (7%)	1 (2%)	
PK‐INR							
	1.0 (0.9–1.1)	1.0 (0.9–1.1)	1.0 (0.9–1.0)	0.737 (0.210–3.019)	1.0 (0.9–1.1)	1.0 (1.0–1.0)	0.665 (0.152–3.327)
Missing	17 (4%)	15 (5%)	2 (4%)		14 (4%)	3 (6%)	
APTT (s)							
	27 (26–29)	27 (26–30)	27 (26–29)	0.201 (0.891–1.025)	27 (26–30)	27 (26–29)	0448 (0.905–1.045)
Missing	18 (5%)	17 (5%)	1 (2%)		15 (4%)	3 (6%)	
Sodium (mmol/L)							
	139 (138–141)	139 (138–141)	139 (138–141)	0.506 (0.867–1.073)	139 (138–141)	139 (138–141)	0.628 (0.917–1.154)
Missing	0 (0%)						
Potassium (mmol/L)							
	4.0 (3.8–4.2)	4.0 (3.8–4.2)	4.0 (3.8–4.2)	0.886 (0.473–2.379)	4.0 (3.8–4.2)	4.0 (3.8–4.2)	0.565 (0.548–3.002)
Missing	1 (0.3%)	1 (0.3%)	0 (0%)		1 (0.3%)	0 (0%)	
CRP (mg/L)							
	3 (1–8)	3 (1–8)	3 (1–7)	0.979 (0.980–1.021)	3 (1–7)	3 (2–7)	0.310 (0.948–1.017)
Missing	72 (19%)	66 (20%)	6 (11%)		66 (20%)	6 (13%)	
Bilirubin (μmol/L)							
	6 (5–9)	6 (5–9)	7 (5–9)	0.705 (0.926–1.053)	6 (5–9)	6 (5–9)	0.258 (0.869–1.038)
Missing	14 (4%)	11 (3%)	3 (6%)		11 (3%)	3 (6%)	
ALT (μkat/L)							
	0.34 (0.26–0.49)	0.35 (0.27–0.52)	0.31 (0.26–0.43)	**0.040 (0.034–0.923)**	0.34 (0.26–0.48)	0.37 (0.26–0.51)	0.659 (0.421–3.930)
Missing	3 (0.8%)	3 (0.9%)	0 (0%)		2 (0.6%)	1 (2%)	
AST (μkat/L)							
	0.39 (0.33–0.48)	0.39 (0.33–0.48)	0.38 (0.32–0.46)	0.206 (0.029–2.141)	0.39 (0.33–0.47)	0.395 (0.34–0.51)	0.142 (0.671–16.086)
Missing	20 (5%)	20 (6%)	0 (0%)		18 (5%)	2 (4%)	
Creatinine (μmol/L)							
	70 (59–82)	69 (59–81)	72 (60–86)	0.294 (0.993–1.024)	70 (59–82)	67 (57–85)	0.991 (0.983–1.017)
Missing	0 (0%)						
Zink (μmol/L)							
	11 (10–13)	11 (10–13)	11 (10–13)	0.846 (0.849–1.222)	11 (10–13)	11 (10–12)	0.285 (0.757–1.085)
Missing	136 (35%)	112 (33%)	24 (45%)		123 (36%)	13 (27%)	
Platelet count (*n* × 10^9^)							
	248 (212–294)	248 (210–291)	259 (220–300)	0.521 (0.997–1.005)	247 (210–292)	262 (227–295)	0.349 (0.998–1.006)
Missing	7 (2%)	7 (2%)	0 (0%)		6 (2%)	1 (2%)	
Leucocyte count (*n* × 10^9^)							
	7.1 (5.8–8.4)	7.1 (5.8–8.4)	6.9 (5.8–8.2)	0.772 (0.907–1.075)	7.1 (5.8–8.4)	7.3 (6.2–8.4)	0.846 (0.916–1.074)
Missing	3 (0.8%)	2 (0.6%)	1 (2%)		3 (0.9%)	0 (0%)	
Peth (μmol/L)							
	0.04 (0.04–0.28)	0.04 (0.04–0.33)	0.04 (0.04–0.2)	0.543 (0.342–1.760)	0.04 (0.04–0.25)	0.04 (0.04–0.33)	0.564 (0.558–2.910)
Missing	228 (59%)	206 (62%)	22 (42%)		197 (58%)	31 (65%)	
Transthyretin (g/L)							
	0.27 (0.23–0.31)	0.27 (0.23–0.31)	0.27 (0.23–0.31)	0.856 (0.010–44.914)	0.27 (0.23–0.31)	0.27 (0.22–0.30)	0.470 (0.002–19.057)
Missing	80 (21%)	69 (21%)	11 (21%)		74 (22%)	6 (13%)	
GT (μkat/L)							
	0.51 (0.33–0.82)	0.51 (0.34–0.81)	0.49 (0.28–0.91)	0.561 (0.615–1.302)	0.50 (0.33–0.81)	0.59 (0.35–0.88)	0.253 (0.891–1.549)
Missing	8 (2%)	7 (2%)	1 (2%)		6 (2%)	2 (4%)	

*Note:* Statistically significant results are presented in bold for increased visibility.

**TABLE 2:** Univariate analyses of preoperative surgical variables and peri‐ and postoperative variables.

Variable	All *n* = 388 (100%)	No flap compromise *n* = 335 (86%)	Flap compromise *n* = 53 (14%)	*p* (95% CI of OR)	No systemic complication *n* = 340 (88%)	Systemic complication *n* = 48 (12%)	p‐value (95% CI of OR)
T‐stage							
1–2	109 (28%)	94 (28%)	15 (28%)	Reference	94 (28%)	15 (31%)	Reference
3–4	217 (56%)	186 (56%)	31 (59%)	0.898 (0.537–2.030)	189 (56%)	28 (58%)	0.829 (0.473–1.822)
N/A	62 (16%)	55 (16%)	7 (13%)	—	57 (17%)	5 (10%)	—
Missing	0 (0%)						
Previous head/neck cancer							
Yes	166 (43%)	138 (41%)	28 (53%)	0.114 (0.894–2.860)	144 (43%)	22 (46%)	0.648 (0.628–2.114)
Missing	0 (0%)						
Previous radiotherapy							
Yes	157 (41%)	130 (39%)	27 (51%)	0.097 (0.915–2.930)	135 (40%)	22 (46%)	0.419 (0.700–2.360)
Missing	0 (0%)						
Indication for surgery							
Primary	224 (58%)	199 (60%)	25 (48%)	Reference	199 (59%)	25 (52%)	Reference
Salvage	127 (33%)	104 (31%)	23 (43%)	0.071 (0.953–3.253)	108 (32%)	19 (40%)	0.303 (0.738–2.658)
Other	37 (10%)	32 (10%)	5 (10%)	0.678 (0.444–3.485)	33 (10%)	4 (8%)	0.965 (0.315–2.951)
Missing	0 (0%)						
Type of free flap							
Radial	162 (42%)	150 (45%)	12 (23%)	Reference	143 (42%)	19 (40%)	Reference
Fibula	120 (31%)	100 (30%)	20 (38%)	**0.018 (1.170–5.341)**	102 (30%)	18 (38%)	0.422 (0.664–2.658)
Scapula	37 (10%)	30 (9%)	7 (13%)	**0.038 (1.061–8.018)**	32 (9%)	5 (10%)	0.764 (0.409–3.384)
Other (single/combined flaps)	69 (18%)	55 (16%)	14 (26%)	**0.006 (1.387–7.302)**	63 (19%)	6 (13%)	0.499 (0.273–1.880)
Missing	0 (0%)						
Type of free flap							
Radial	162 (42%)	150 (45%)	12 (23%)	Reference	143 (42%)	19 (40%)	Reference
All other (single/combined)	226 (58%)	185 (55%)	41 (77%)	**0.003 (1.406–5.459)**	197 (58%)	29 (60%)	0.745 (0.598–2.054)
Missing	0 (0%)						
Duration of surgery (h)							
	9.5 (8.0–11.0)	9.3 (7.9–10.5)	10.8 (9.0–12.0)	**< 0.001 (1.119–1.416)**	9.3 (8.0–10.8)	10.0 (8.3–11.5)	0.370 (0.936–1.195)
Missing	0 (0%)						
Perioperative blood loss (mL)							
	300 (200–600)	300 (200–550)	400 (225–700)	**0.017 (1.000–1.002)**	300 (200–600)	300 (200–575)	0.490 (0.999–1.001)
Missing	1 (0.3%)	1 (0.3%)	0 (0%)		1 (0.3%)	0 (0%)	
Red blood cell transfusion (< 48h)							
Yes	161 (41%)	125 (37%)	36 (68%)	**< 0.001 (1.918–6.599)**	138 (41%)	23 (48%)	0.336 (0.734–2.469)
Missing	0 (0%)						
Transfused units of red blood cells (*n*)							
	0 (0–1)	0 (0–1)	1 (0–2)	**0.001 (1.152–1.721)**	0 (0–1)	0 (0–2)	0.681 (0.830–1.331)
Missing	0 (0%)						
Perioperative use of vasopressors or inotropy							
Any	294 (76%)	250 (75%)	44 (83%)	0.189 (0.779–3.547)	259 (76%)	35 (73%)	0.622 (0.425–1.668)
Missing	0 (0%)						
Length of stay ICU (h)							
	18 (16–21)	18 (16–20)	19 (17–34)	**0.006 (1.006–1.036)**	18 (16–21)	18 (16–21)	**0.039 (1.001–1.028)**
Missing	1 (0.3%)	1 (0.3%)	0 (0%)		1 (0.3%)	0 (0%)	
Fluid balance 24 h (mL)							
	3400 (2400–4400)	3400 (2400–4400)	3400 (2200–4400)	0.931 (1.000–1.000)	3300 (2300–4300)	3800 (2700–4800)	0.079 (1.000–1.000)
Missing	16 (4%)	15 (5%)	1 (2%)		16 (5%)	0 (0%)	
Magnesium substitution (mmol)							
	10 (0–20)	9 (0–20)	17 (0–20)	0.110 (0.996–1.035)	8 (0–20)	19 (0–20)	**0.031 (1.002–1.042)**
Missing	7 (2%)	7 (2%)	0 (0%)		7 (2%)	0 (0%)	
Phosphate substitution (mmol)							
	0 (0–8)	0 (0–7)	4 (0–10)	0.481 (0.991–1.020)	0 (0–7)	1 (0–13)	0.119 (0.997–1.024)
Missing	7 (2%)	7 (2%)	0 (0%)		7 (2%)	0 (0%)	
Postoperative flap monitoring							
Cook electrode or microdialysis	313 (81%)	273 (82%)	40 (76%)	Reference	278 (82%)	35 (73%)	Reference
Visual inspection	73 (19%)	62 (19%)	11 (21%)	0.603 (0.588–2.493)	60 (18%)	13 (27%)	0.126 (0.859–3.448)
Missing	2 (0.5%)	0 (0%)	2 (4%)		2 (0.6%)	0 (0%)	

*Note:* Statistically significant results are presented in bold for increased visibility.

#### 3.1.1 Preoperative Variables

The median age at surgery was 66 years (56–71). For flap compromise, no association was shown with age, whereas increased systemic complications were seen with increasing age (*p* = 0.021). The ratio of male to female patients was 236:152 (61% vs. 39%) and there was no association between sex and either type of complication. Neither were any associations seen between complications and smoking or alcohol overconsumption (defined as a weekly intake of more than 14 standard units for males and 9 standard units for women).

No single comorbidity was associated with either flap compromise or systemic complications. Neither were any of the tested risk prediction instruments associated with flap compromise. However, for systemic complications, all instruments showed significant associations. In this group, the median ASA‐PS class was 3 (2–3), as compared to 2 (2–3) in patients without systemic complications (*p* = 0.002). For CCI, the median score was 3 (2–4) for patients affected by complications, while the non‐complication group had a median score of 2 (1–3) (*p* = 0.005). When using the HN‐CCI, 18 patients (38%) with systemic complications had a score of ≥ 1, compared to 67 (20%) in the group without complications (*p* = 0.006). Finally, ACS‐NSQIP had a similar association with a median risk of serious complication of 18% in the systemic complication group versus 15% in unaffected patients (*p* = 0.006).

Only two of the preoperative biochemical analyses (see complete data in Table 1) demonstrated an association with complications: lower albumin linked to postoperative systemic complications (*p* = 0.026) and lower alanine aminotransferase (ALT) linked to flap compromise (*p* = 0.040).

There was no association between surgical indication and either type of postoperative complications; neither could any link be seen between previous radiotherapy, previous HNC, or T‐stage of the current tumor and complication rates.

#### 3.1.2 Peri‐ and Postoperative Variables

For peri‐ and postoperative variables, the associations with flap compromise predominated. Increased risk of flap compromise was seen when using any other flap than the radial forearm (*p* = 0.003), as well as with increased duration of surgery (*p* < 0.001).

The median blood loss during surgery was 300 mL (200–550) in the group with no flap compromise versus 400 mL (225–700) in the group with compromised flaps (*p* = 0.017). More patients with flap compromise received red blood cell transfusion within the first 48 h (*p* < 0.001).

Magnesium substitution during the day of surgery was more frequent among patients with systemic complications (*p* = 0.031).

No differences in any complication rates were seen in relation to perioperative use of vasopressors or inotropy, or postoperative fluid balance.

## 4 Multivariable Analyses

For detailed presentation of multivariable regression analyses, see Table 3.

**TABLE 3:** Multivariable analyses of factors associated with flap compromise and systemic complications respectively.^a^

Independent variables	OR	95% CI of OR	*p‐value*
Flap compromise^b^			
Surgery time (h)	1.192	1.054–1.347	**0.005**
Red blood cell transfusion < 48 h	2.835	1.493–5.384	**0.001**
Systemic complications^c^			
ASA‐PS	2.059	1.175–3.609	**0.012**
Albumin preop (g/L)	0.935	0.871–1.004	0.066
Mg substitution (mmol)	1.022	1.001–1.043	**0.036**
Systemic complications^d^			
CCI	1.285	1.038–1.590	**0.021**
Albumin preop (g/L)	0.928	0.863–0.997	**0.041**
Mg substitution (mmol)	1.019	0.999–1.040	0.064
Systemic complications^e^			
HN‐CCI (≥ 1)	2.169	1.109–4.242	**0.024**
Albumin preop (g/L)	0.927	0.863–0.996	**0.038**
Mg substitution (mmol)	1.021	1.000–1.042	**0.047**
Systemic complications^f^			
NSQIP serious complications	1.069	0.993–1.151	0.074
Albumin preop (g/L)	0.933	0.868–1.003	0.062
Mg substitution (mmol)	1.021	1.001–1.042	**0.042**

^a^ Only the final step of the backward Wald method is presented.

^b^ Variables included: surgery time (h), red blood cell transfusion within 48 h, blood loss, previous radiotherapy, flap type.

^c^ Variables included: ASA, age, preoperative albumin (g/L), Mg substitution (mmol).

^d^ Variables included: CCI, preoperative albumin (g/L), Mg substitution (mmol).

^e^ Variables included: HN‐CCI, age, preoperative albumin (g/L), Mg substitution (mmol).

^f^ Variables included: NSQIP serious complications, age, preoperative albumin (g/L), Mg substitution (mmol).

Collinearity for flap compromise was detected between perioperative blood transfusion and number of transfused units. For systemic complications, collinearity was detected between age and CCI score and ASA‐PS class and NSQIP risk of serious complications, respectively. To avoid any interaction between risk prediction instruments (despite lack of collinearity) no multivariable analyses included more than one such instrument score.

For flap compromise, the independent variables included in the model were previous radiotherapy, type of free flap, surgery time, perioperative blood loss, and red blood cell transfusion. Surgery time (OR 1.192 (1.054–1.347), *p* = 0.005) and red blood cell transfusion (OR 2.835 (1.493–5.384), *p* = 0.001) were factors independently associated with flap compromise.

For systemic complications, several models were tested, including age, preoperative albumin, and perioperative magnesium substitution, combined with the different risk prediction instruments (complete presentation in Table 3). The statistical significance varied for preoperative albumin and magnesium substitution between regression models, while all risk prediction instruments except for NSQIP remained predictive in their respective models (ASA‐PS OR 2.059 (1.175–3.609), *p* = 0.012; CCI OR 1.285 (1.038–1.590), *p* = 0.021 and HN‐CCI score ≥ 1 OR 2.169 (1.109–4.242), *p* = 0.024).

## 5 Discussion

Currently, we lack robust tools to guide treatment recommendations about extensive reconstructive surgery for individual patients, and the aim of this registry‐based population study was to contribute to the facilitation of multidisciplinary decision making and patient communication concerning perioperative risks. Our study, including 388 consecutive patients subjected to head and neck free flap surgery, found that several pre‐ and perioperative variables were associated with flap compromise and systemic complications.

In microvascular surgery, the survival of a well‐functioning flap is a keystone to restoring function, and our study showed several factors associated with the risk of flap compromise. The choice of any other type of flap than the radial was associated with an increased risk of postoperative compromise, even more so if rarer or multiple flaps were used. This is in contradiction with several previous studies, where no difference has been seen between types of flaps [30, 31], but in line with D'Andréa and colleagues who found more complications in patients undergoing osseous reconstruction than in soft tissue reconstruction [21]. The radial flap, being by far the most used in our material, could contribute to the results, but the fact that flap type might affect surgical outcome makes the choice—if possible—worthy of careful consideration.

Longer surgery time was another factor correlated with flap compromise. Extended surgery time could be attributed to complex resections and reconstructions, but also to immediate problems with the flap and efforts to save it. However, the link between surgery time and flap complications has been shown in several previous studies [32, 33].

Other factors correlated with flap compromise in our study were perioperative bleeding and red blood cell transfusion, where only the latter remained associated in the multivariable regression analysis. Previous studies have shown similar results [29, 34], and Patil et al. even showed decreased overall survival in head and neck free flap patients receiving red blood cell transfusion, despite adjusting for factors such as age and oncologic stage [35]. It remains unclear whether this effect is attributable to the still not fully understood immunological effects of red blood cell transfusion [36], or other factors. Further research is needed to define a safe hemoglobin threshold in this patient population, but given that only transfusion and not bleeding volume remained correlated with flap compromise after adjusting for surgery time and flap type, the data from the current study supports a restrictive transfusion policy.

While no isolated comorbid condition predicted systemic complications, ASA‐PS, CCI, and HN‐CCI all were associated with increased systemic complications in both univariate and multivariable analyses, indicating that the total burden of comorbidities is the important factor, rather than any single organ‐specific disease. This is in line with many previous studies, although differing results have been seen for the different instruments [16]. For HNC patients, CCI is one of the predominantly used and well‐validated instruments [5], and NSQIP has, in recent years, gained increasing attention, especially among anesthesiologists, as specifically designed for the perioperative setting. However, they are both lengthy and complex and, therefore, impractical in the hectic clinical reality. Speed and ease of completion are factors shown to be important for the actual implementation of risk prediction instruments [15]. Our data do not indicate any advantages of the more complex CCI and NSQIP, which supports the use of the simpler ASA‐PS and HN‐CCI scores. To our knowledge, this study is the first to show HN‐CCI as a predictor specifically for systemic complications after head and neck free flap surgery.

In our study, increasing age was associated with increased risk of systemic complications in univariate analysis, but the association was eliminated in the multivariable analyses, indicating that the comorbidities are more important than age itself. In previous publications the effect of age on outcome varies [18, 32, 37], and larger meta‐analyses are likely needed to determine any actual effect of age on complication risks.

Another factor associated with systemic complications was preoperative albumin levels. The rationale of hypoalbuminemia as a surrogacy of malnutrition is in line with results from several other studies [13, 27, 38]. This finding is, in our view, particularly interesting, as albumin levels are at least partially accessible to intervention, something that has been evaluated by Xu and colleagues [39] with perioperative albumin substitution; however, with positive effects on flap survival rather than systemic complications.

In the present study, perioperative magnesium substitution was linked to systemic complications. One possible explanation for this finding is that magnesium supplementation may be related to intraoperative cardiovascular instability, with arrhythmia tendency being a common indication for magnesium substitution. However, the effects of magnesium itself, such as vasodilation, cannot be excluded as a cause of complications, and the finding encourages further investigation.

Different parameters increasing the risks of different complications make the preoperative risk prediction challenging, but it is our belief that this reflects the complexity of the patient group and treatment course. It further strengthens our conviction of the importance of multidisciplinary cooperation, in which anesthesiologists play an important role throughout the entire perioperative phase. The evolution of artificial intelligence (AI) has introduced huge and unprecedented possibilities for data merging and creation of prediction models, but however promising the practical applicability of AI is, it is still limited [40] and judicious clinical assessments are likely to still be vital for the foreseeable future.

As is common with retrospective studies, limitations include missing data and subjective reporting for certain variables, such as alcohol consumption and socioeconomic factors. The selection of independent variables recorded also warrants discussion. Given an aging patient population, incorporating a frailty scale would in retrospect have been of interest; however, such scales were not widely established when our database was initiated. Factors concerning specific surgical aspects, such as surgeon experience and type of vessel anastomoses, would also have been of interest with regards to flap compromise.

A strength in this study is the population‐based cohort with consecutive patients and a small team involved in patient care as well as study execution, facilitating consistency in both medical treatment and data collection. We believe that the results of our study can enhance the understanding of perioperative complication risks for patients undergoing major head and neck reconstructive surgery, and hereby contribute to preoperative patient‐doctor discussions as well as decision making. To draw definitive conclusions about predictive factors, further meta‐analyses are necessary, exploring these aspects across diverse settings.

## 6 Conclusion

This registry‐based population study of perioperative risk prediction in patients undergoing major head and neck free flap surgery showed a strong association between surgery time and perioperative red blood cell transfusion and flap compromise. ASA‐PS, CCI, and, for the first time, its simplified version HN‐CCI were shown to be independently associated with systemic complications. Our results support a focus on basics with the use of well‐established, quick, and uncomplicated risk prediction instruments and clinical parameters in the planning and execution of head and neck free flap surgery.

## Author Contributions

K.P.: Conceptualisation, formal analysis, funding aquisition, project administration, writing original draft/revision. M.T.: Data curation, investigation, formal analysis, writing reviewing/editing. L.W.S.: Conceptualisation, project administration, supervision, writing reviewing/editing. T.K.: Conceptualisation, funding aquisition, methodology, project administration, resources, supervision, writing reviewing/editing. C.N.: Validation, supervision, writing reviewing/editing. J.S.: Conceptualisation, formal analysis, project administration, supervision, writing reviewing/editing.

## Conflicts of Interest

The authors declare no conflicts of interest.

## Data Availability

The data that support the findings of this study are available on request from the corresponding author. The data are not publicly available due to privacy or ethical restrictions.
